# Sentinel NOSE: Prospective feasibility study on sentinel lymph node biopsy in bulky nasal vestibule cancer

**DOI:** 10.1371/journal.pone.0325764

**Published:** 2025-06-13

**Authors:** Michal D. Czerwinski, Ellen M. Zwijnenburg, Anne I. J. Arens, Adriana C.H. Van Engen, Jimmie Honings, Willem L.J. Weijs, Robert P. Takes, Johannes H.A.M. Kaanders, Cornelia G. Verhoef

**Affiliations:** 1 Department of Radiation Oncology, Radboud University Medical Center, Nijmegen, The Netherlands; 2 Department of Radiology and Nuclear Medicine, Radboud University Medical Center, Nijmegen, The Netherlands; 3 Department of Pathology, Radboud University Medical Center, Nijmegen, The Netherlands; 4 Department of Otorhinolaryngology, Radboud University Medical Center, Nijmegen, The Netherlands; 5 Department of Oral and Maxillofacial Surgery, Radboud University Medical Center, Nijmegen, The Netherlands; Taichung Veterans General Hospital, TAIWAN

## Abstract

**Objective:**

Patients with bulky squamous cell carcinoma of the nasal vestibule are at high risk of nodal relapse and have worse prognosis, despite modern neck imaging. More sensitive assessment of the neck may obviate elective treatment and ultimately reduce the risk of nodal relapse and improve outcome.

The goal of this study was to evaluate the feasibility, safety and tolerability of sentinel lymph node biopsy (SLNB) in staging of bulky squamous cell nasal vestibule carcinoma (NVC).

**Methods:**

Ten patients with cT1-T2N0 squamous cell NVC with diameter ≥1.5 cm, planned for curative brachytherapy, were prospectively included to undergo four peritumoral injections of ICG-[^99m^Tc]Tc-nanocolloid. Subsequently, dynamic, static and SPECT/CT images were performed. The following day SLNB was conducted under general anesthesia combined with brachytherapy implantation of the primary tumor.

**Results:**

Sentinel lymph nodes were visualized in all patients (median 3.5 per patient), with successful subsequent biopsy in all cases (median yield 2.5 per patient). Occult lymph node metastases were found in 50% of patients. The procedure was well-tolerated.

**Conclusion:**

SLNB in bulky NVC is feasible and safe, with a high success rate of executing the SLN procedure. The relatively high incidence of occult metastases in this population is consistent with earlier findings. Further research is needed to quantify the impact of SLNB in terms of total treatment burden and oncological outcomes.

## Introduction

Squamous cell carcinoma of the nasal vestibule (NVC) is a rare, aggressive and sometimes mutilating cancer [1]. In locally and regionally advanced disease, elective neck treatment is usually recommended to improve regional control [2]. But in early-stage nasal vestibule carcinoma (ES-NVC), management of the neck has been a subject of persistent debate.

Our previous research identified a subset of patients with bulky tumors (≥1.5 cm in diameter and/or ≥2 cm³ in volume) facing a high nodal relapse risk of up to 40% versus approximately 5% for smaller tumors [3,4]. Given that the presence of even a single nodal metastasis decreases survival in head-and-neck cancers (HNC) by approximately 50% [2], it raises consideration for elective neck treatment in bulky disease, either through surgery or irradiation [5,6]. However, in the era of minimally invasive treatments, a novel approach has bridged the gap between imaging and elective treatment of the neck.

Sentinel lymph node biopsy (SLNB) has been a routine diagnostic tool in several cancer types such as breast cancer and melanoma for years, and also gained widespread acceptance as the most accurate neck staging modality in cN0 early-stage oral squamous cell carcinoma (SCC), boasting an excellent negative predictive value of 90–100% [7] and reduced morbidity rates [8,9]. Similarly encouraging results have also been demonstrated in other mucosal HNC [10] and cutaneous SCC [11,12]. Recent advancements, such as the introduction of the hybrid fluorescent-radioactive tracer indocyanine green (ICG)-[^99m^Tc]Tc-nanocolloid, have further improved intra-operative SN identification [13,14].

The purpose of this prospective study was to investigate the clinical and technical feasibility of the SLN procedure for bulky nasal vestibule carcinoma and systematically identify potential obstacles or unexpected phenomena. We hypothesized that SLNB in bulky NVC will prove feasible as in other head-and-neck tumors and, ultimately, enhance regional control rates in patients with early-stage disease without adding significant morbidity.

## Materials and methods

### Patients

**Fig 1:**
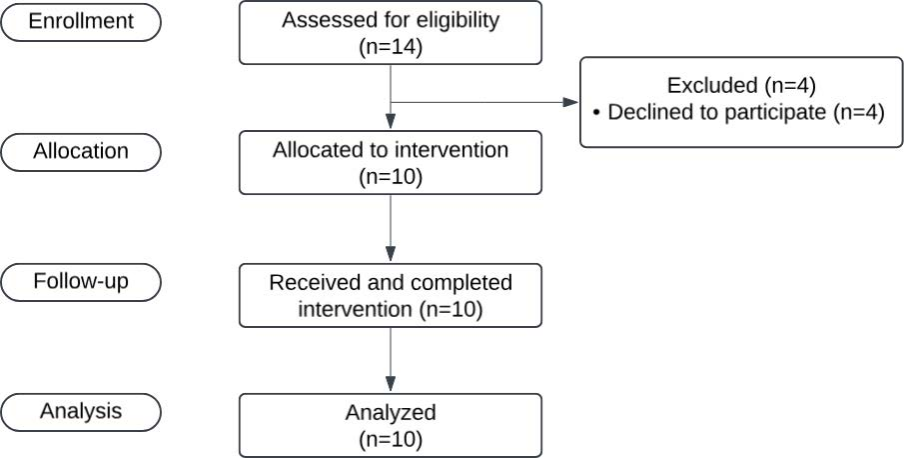
CONSORT diagram.

In this prospective non-randomized study, 10 adult patients with newly diagnosed Wang cT1-T2 squamous cell NVC ≥ 1.5 cm, planned for curative brachytherapy at the Radboudumc Centre for Head‐and‐Neck Oncology were included (Fig 1). Initial disease staging consisted of physical examination of the nasal and head-and-neck region, tumor biopsy, MRI and US(-FNAC) of the neck.

**Fig 2:**
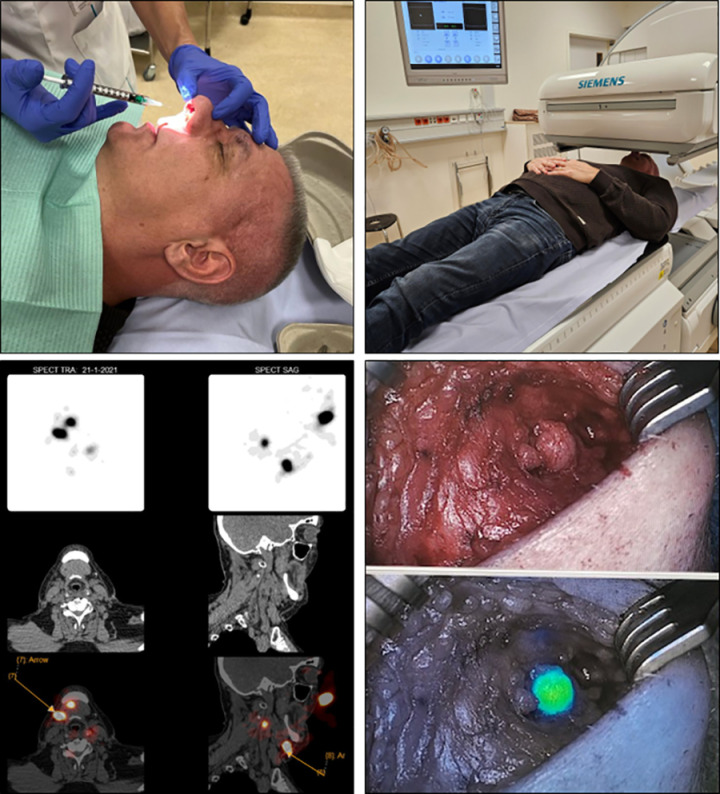
Overview of study procedures (from left to right, top to bottom; written patient permission acquired; the SLN depicted in this figure is in right neck level Ib): peritumoral ICG-[^99m^Tc]Tc-nanocolloid injection, imaging with the SPECT/CT camara, SPECT/CT images, perioperative view without and with immunofluorescence of a sentinel lymph node.

Patients were eligible if they had not undergone neck surgery or irradiation previously, had a WHO performance score of 0, 1, or 2, were clinically negative for neck involvement as determined by physical examination, US-FNAC and MRI and able to provide written informed consent. Regional ethical board approval was obtained (Commissie Mensgebonden Onderzoek Regio Arnhem-Nijmegen dossier number NL70706.091.20). Written informed consent was obtained from all participants, and the individual whose photograph was used in a figure has also given written informed consent. The study was registered in clinicaltrials.gov (NCT05637307). The individual pictured in Fig 2 has provided written informed consent (as outlined in PLOS consent form) to publish their image alongside the manuscript.

Fig 2 provides a pictorial overview of the study procedures. The study protocol is attached as S1 Protocol.

### Injection technique

Thirty to sixty minutes prior to the tracer injection, patients received topical anaesthesia by uni- or bilateral infraorbital nerve block with articain 4%/adrenalin 5 ug/ml depending on tumor location and/or nasal gauzes drenched in lidocaine 10%. Additionally, starting from the second study patient, gauzes were placed posterior to the tumor in the nasal passage to avoid leakage of the radiopharmaceutical in the nose and subsequently the nasopharynx, airway and esophagus.

Subsequently, four peritumoral injections of 20MBq ICG-[^99m^Tc]Tc-nanocolloid (Nanocoll, GE Healthcare, Eindhoven, The Netherlands) were administered by the treating radiation oncologist and nuclear physicist.

### Preoperative imaging and interpretation

Immediately after injection, dynamic images of the neck in anterior view for 10 minutes and early static images in anterior and both lateral views were acquired. After two to four hours, late static views in anterior and both lateral views and single-photon emission computed tomography and computed tomography (SPECT/CT) of the head-and-neck region were performed. Assessment of the images, to identify and localize SLNs, was carried out by an experienced head and neck cancer nuclear medicine physician (AA) according to current EANM guidelines [15] and in consensus with the radiation oncologist (EZ, LV) and head-and-neck surgeon (RT, JH). Sentinel lymph node locations were documented using neck levels as defined by the American Head and Neck Society and Grégoire et al [16,17] and marked on the skin using the camera. The study protocol specified that retropharyngeal, buccal or pre-auricular and level III-VI SLNs would not be surgically removed if higher echelon sentinel lymph nodes were present following a consensus decision, due to the heightened risk of associated morbidity.

### Surgical procedure

The surgical SLNB procedure was conducted in combination with catheter placement for brachytherapy under general anesthesia on the next morning following tracer injection. Incisions were chosen taking into account the potential need for a second procedure such as neck dissection. Sentinel lymph nodes were located using a gamma probe, fluorescence camera and anatomical localization based on SPECT/CT-imaging, and subsequently removed. When necessary, a drain was left in place, and the incision was closed in layers. The total amount of counts in 10 seconds as measured by the gamma probe and the presence or absence of fluorescence of the removed lymph nodes were noted. Brachytherapy catheters were then placed in the usual manner for treatment of the primary tumor [4].

### Pathology

Pathological assessment involved serial step sectioning at 5 levels with 200 µm intervals and staining with hematoxylin-eosin and pan-cytokeratin antibody. Metastases were categorized as isolated tumor cells (≤0.2 mm), micro-metastases (>0.2 mm and ≤ 2 mm) or macro-metastases (>2mm). Additionally, the presence of extra-capsular extension was documented if observed.

### Clinical consequences

In case of tumor negative SLNs (free of tumor cells), no additional treatment of the neck was required. Additional staging information in form of N0(sn) was documented and patients were planned to undergo standard treatment and follow-up consisting of periodic physical examination for a total of 3–5 years. In the event of a tumor positive SLN, additional selective neck node dissection of level I-III was performed of either the involved side in case of a lateralized tumor, or bilaterally in case of a midline tumor, according to existing guidelines on SLNB in oral cancer [18].

### Endpoints

The primary endpoint was successful identification of SLNs assessed by imaging. Success was defined by localization of one or more SLNs in a minimum of 7 out of 10 patients, taking an initial learning curve into account.

Secondary endpoints included tolerability and safety of the procedure measured by the incidence of adverse events associated with peritumoral tracer injection and pain score on a visual analogue scale ranging 1–10 immediately post-tracer injection, analgesic usage during and after injection, surgical sentinel lymph node yield (considered successful with at least one histopathologically investigated sentinel lymph node), the presence of tumor positive SLNs and the occurrence of peri- and postoperative complications according to the Clavien-Dindo classification related to the SLNB [19].

### Statistical analysis

Mostly descriptive statistics were employed due to the explorative nature of the study using IBM SPSS 27. No power calculation was performed, as the study did not focus on quantitative clinical outcomes.

## Results

Between 12 January 2021 and 3 November 2023, ten patients were prospectively included (Table 1). The study procedures did not cause delay in start of cancer treatment, and the median time between staging and study procedure/start of treatment was 17.5 days (range 7–30).

**Table 1:** Overview of patient characteristics and outcomes (neck node levels according to Robbins and Gregoire et al [16,17]).

Patient no.	Age, sex	Tumor stage^*^ and diameter	Primary laterality and locations	No. of SLN identified on images	No. of SLN harvested	No. and anatomic level of metastases	Additional neck dissection	Yield in neck dissection
1.	76, M	T2, 2.5 cm	Right Dome+ala+floor	10	4	**1 (micro) ipsilateral level Ia**	Level I-III bilaterally	0/81 nodes positive
2.	65, M	T1, 1.5 cm	Left Ala	4	2 (+1 non-SLN)	–	–	–
3.	66, F	T1, 1.6 cm	Bilateral Dome+septum	8	6 (+1 non-SLN)	–	–	–
4.	86, M	T2, 2.5 cm	Left Dome+septum+floor	5	3 (+2 non-SLN)	**1 (macro) ipsilateral level Ib**	Level I-III bilaterally	0/64 nodes positive
5.	68, F	T2, 3.0 cm	Bilateral Septum+floor+philtrum	4	4	–	–	–
6.	75, M	T1, 3.0 cm	Left Dome+ala+floor	3	2 (+1 non-SLN)	**1 (macro) ipsilateral level Ib + 1 metastasis level III of unrelated second tumor**†	Level I-V ipsilaterally	**1/60 nodes positive for squamous cell carcinoma**‡
7.	52, M	T2, 2.5 cm	Bilateral septum Left dome+floor	3	1 (+1 non-SLN)	**1 (macro with extranodal extension), left level Ib** ^**3**^	Level I-V left	0/26 nodes positive‡
8.	76, M	T1, 1.5 cm	Left Septum+floor	3	3	–	–	–
9.	60, M	T2, 3.0 cm	Bilateral Dome+ala+septum	1	1	**1 (macro) left level Ib**	Level I-III bilaterally	0/45 nodes positive
10.	63, M	T1, 1.5 cm	Bilateral septum Left ala	3	2 (+1 non-SLN)	–	–	–

* According to Wang classification.

† An unexpected metastasis of an occult concurrent thyroid cancer was found.

‡ Indication for post-operative radiotherapy.

The four peritumoral tracer injections were all successful and generally well-tolerated by all patients. The pain post-injection was transient. Directly after the injection, the mean pain score was 4.4 (range 2–9), which decreased to 0.2 (range 0–2) within five minutes (Table 2). Notably, no additional analgesics were required post-injection.

**Table 2:** Pain scores (numerical 0–10 scale) per patient after tracer injection.

Patient no.	Age, sex	Tumor stage and diameter	Primary laterality and locations	Anaesthesia type	Directly after injection	5 minutes after injection	10 minutes after injection
1.	76, M	T2, 2.5 cm	Right Dome+ala+floor	Infraorbital block + xylocaine gauzes	2	0	0
2.	65, M	T1, 1.5 cm	Left Ala	Infraorbital block + xylocaine gauzes	3	0	0
3.	66, F	T1, 1.6 cm	Bilateral Dome+septum	Infraorbital block + xylocaine gauzes	3	0	0
4.	86, M	T2, 2.5 cm	Left Dome+septum+floor	Infraorbital block + xylocaine gauzes	4	0	0
5.	68, F	T2, 3.0 cm	Bilateral Septum+floor+philtrum	Infraorbital block + xylocaine gauzes	6	0	0
6.	75, M	T1, 3.0 cm	Left Dome+ala+floor	Infraorbital block + xylocaine gauzes	9	2	0
7.	52, M	T2, 2.5 cm	Bilateral septum Left dome+floor	Infraorbital block + xylocaine gauzes	7	0	0
8.	76, M	T1, 1.5 cm	Left Septum+floor	Infraorbital block + xylocaine gauzes	2	0	0
9.	60, M	T2, 3.0 cm	Bilateral Dome+ala+septum	Infraorbital block + xylocaine gauzes	2	0	0
10.	63, M	T1, 1.5 cm	Bilateral septum Left ala	Xylocaine gauzes	6	0	0
Mean					**4,4**	**0,2**	**0**

Imaging procedures adhered to the study protocol and were successful in all ten patients. The median number of imaged SLNs was 3.5 per patient (range 1–10).

**Fig 3:**
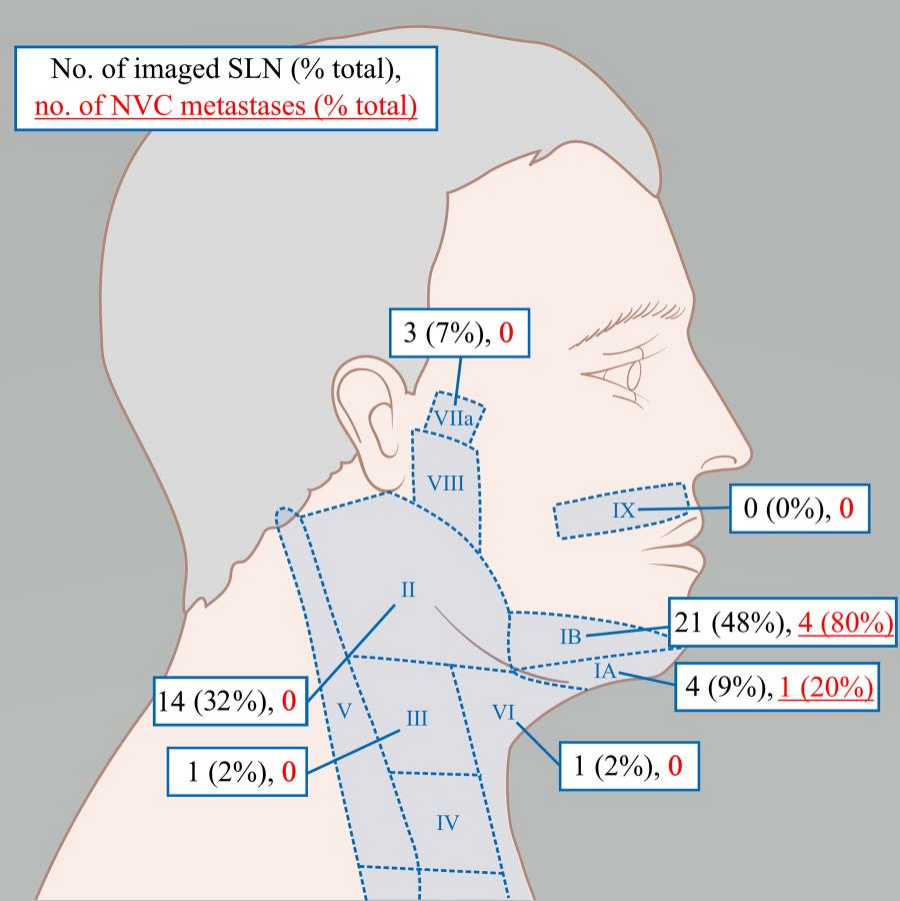
Anatomical overview of imaged sentinel lymph nodes and histology confirmed metastases (neck levels according to Robbins and Gregoire et al; VIIa; retropharyngeal, VIII; preauricular, IX; buccofacial [16,17]). Adapted from Cancer Research UK/ Wikimedia Commons under the Creative Commons Attribution-Share Alike 4.0 International license.

Most SLNs were identified in level Ib (21 SLNs, 48%), level II (14 SLNs, 32%) and level Ia (4 SLN’s, 9%). Notably, no SLNs were found in the buccal or pre-auricular regions. Lower echelon (levels III-VI) and retropharyngeal SLNs were uncommon; 3 nodes (7%) were found retropharyngeally, and 1 in level III and VI each (2%). Detailed individual patient characteristics and SLN locations are presented in Fig 3.

Sentinel lymph nodes were surgically removed in all 10 patients, with a median yield of 3 per patient (range 1–6). Out of 44 SLNs identified on imaging, 28 were harvested (64%). Five (11%) contralateral SLNs were not removed by consensus due to high suspicion of contralateral tracer diffusion in a clearly lateralized tumor in patient 1, four (9%) were not removed due to retropharyngeal or level VI localization and seven (16%) could either not be identified intraoperatively, or were removed but did not contain ICG-[^99m^Tc]Tc-nanocolloid and were therefore not registered as SLNs after surgery.

One patient (patient 7) had a clearly pathologically enlarged SLN on SPECT/CT. This SLN could not be identified during surgery using the gamma probe and near-infrared fluorescence camera, but was found and removed guided by the localisation on pre-operative imaging. Since one of the inclusion criteria of the study was cN0 status, a thorough review of the initial staging was undertaken. This SLN was not palpable, but was pathologically enlarged on initial staging MRI (14x19mm). However, it was initially interpreted as a reactive lymph node and not considered malignant due to extensive bilateral lymphadenopathy and diffuse sinonasal mucosal swelling observed on initial MRI and CT. This conclusion was reinforced by two negative US-FNAC results, attributing the changes to a likely sinonasal infection. Further scrutiny, including a re-evaluation of the cytology, did not alter these findings.

Sentinel lymph node metastases were found in 5 out of 10 patients. These comprised one microscopic and four macroscopic metastases (of 0.3 cm, 0.5 cm, 1.5 cm and 2 cm with extranodal extension). All NVC metastases were found in either level Ib (80%) or level Ia (20%) as presented in Fig 3. In addition, one patient had an unexpected SLN metastasis from an occult concurrent thyroid cancer in level III. Detailed individual SLN locations can be found in S2 Table.

All patients with nodal metastases had tumors of at least 2.5 cm in diameter and Wang stage T2 tumors. All underwent additional neck dissection as per study protocol, which was bilateral if the midline was compromised. In one case (patient 6), an additional metastasis without ENE was discovered in the neck dissection specimen (Table 1). Subsequently, adjuvant neck radiation was proposed due to a total of 2 positive nodes but was refused by the patient. Patient 7 underwent adjuvant neck radiation because of extranodal extension. In the remaining three patients, no additional metastases were discovered in the neck dissection specimen and therefore did not receive adjuvant neck radiation.

## Discussion

To our knowledge, this is the first study documenting the application of SLNB in (bulky) NVC, providing valuable insights into the procedure and clinical and technical feasibility in this tumor type.

The first challenge was anesthesia of the nasal area, known for its complexity. The combination of an infraorbital block with intracavital lidocaine gauzes effectively managed the pain associated with tracer injection, although transient discomfort was still reported. No additional complaints were noted after the injection. We conclude from the rapid decrease in pain following the injection, combined with the absence of a need for additional post-procedure analgesia, that the procedure was well-tolerated.

Despite extensive experience with SLNB for oral tumors at our center, a learning curve was observed for this new subsite. Particularly in the first patient, nasopharyngeal contamination resulting in a very high number of sentinel nodes on imaging highlighted the need for proficiency and carefulness in injecting.

The imaging procedure was successful in all ten patients, and also at least one SLN was successfully harvested in all of our cases. This high success rate corresponds with SLN detection rates observed in other head and neck subsites such as the lip (90–100%) [20], oral cavity (92–100%) [7], pharynx (96–100%), larynx and hypopharynx (87–100%) [10] and can partly be attributed to the use of a dual labeled tracer approach which is known for improved accuracy over single-labeled tracer methods [13,14]. However, this positive result still has to be interpreted with caution due to the small sample size of ten.

There was one patient experiencing transient paresis of the marginal branch of the facial nerve after surgery. This was considered mild and acceptable. No major SLNB related complications were observed.

A notable case was patient 7, where the reduced accuracy of the SLNB procedure in macroscopic node-positive disease became evident. The disruption of normal tracer dynamics in nodes mostly replaced with tumor can result in re-routing of tracer fluid, sometimes falsely omitting nodes with gross disease, or allowing only for limited tracer accumulation [21–23]. This phenomenon is present in all tumor types where SLNB procedures are applied, and is not specific for NVC. Its occurrence highlights the fact that a clinically suspect node should always be excised, even in the case of tracer and fluorescence negativity.

Although no definitive conclusions can be drawn from this small feasibility study, interestingly, the high incidence of occult metastases in 50% of patients with bulky NVC corroborates previous findings in retrospective cohorts, particularly the 40% nodal recurrence risk in bulky disease [3,4]. Notably, elective neck treatment is generally not advocated in ES-NVC [24], as literature reports overall nodal recurrence rates ranging from 0% to 29% in ES-NVC [25–28]; therefore the observed trend of high incidence of occult metastases appears to be specific for bulky NVC. In context, a recent large meta-analysis on diagnostic accuracy of SLNB in mucosal head-and-neck cancers found a pooled prevalence of clinically occult metastases of 30% in oropharyngeal- and laryngeal/hypopharyngeal cancer [10]. However, this analysis included T1-T4 tumors without distinguishing between bulky and non-bulky disease, limiting direct comparisons.

It is also important to note that the current study was not powered to investigate this outcome thoroughly, and as such, these findings should be interpreted only as an observed trend.

Another notable observation was that all patients with metastases had tumors larger than 2.5 cm in diameter. Since the recognition of NVC as a separate subsite, suggestions are done to update and improve current staging systems [29]. We advocate to futher investigate the prognostic relevance of tumor size, drawing parallels with other head and neck cancers such as the lip and oral cavity.

There are clear limitations to the study. The small size of the cohort, the limited follow-up and the study’s exploratory nature leaves relevant questions open, such as the generalizability of these results and ultimate regional control rate after SLNB, and also which therapy – if any- is optimal after a positive SLNB. NVC is a rare cancer with an estimated incidence of 50–60 patients/year in the entirety of the Netherlands, which imposed a practical limit on a feasible number of study participants in a single center, in a limited amount of time. Especially because we aimed to include only patients with large tumors. In our study, all patients with a positive SLNB underwent therapeutic selective neck dissection with adjuvant radiotherapy when indicated. Future research should clarify if this procedure is successful in improving clinical outcomes such as ultimate regional control, as well as reducing the number of patients receiving extensive multimodality treatment.

In the standard approach for head and neck cancers, a SLN-procedure serves as a treatment de-escalating tool, by forgoing elective neck dissection and/or irradiation in patients with negative SLN. In NVC, there is usually no elective treatment of the clinically negative neck, and salvage in case of metachronous metastases will likely consist of bilateral neck dissection often followed by postoperative radiotherapy. In our study, only two of the five patients with tumor-positive SLNs needed adjuvant postoperative radiotherapy, suggesting a reduced treatment burden, and therefore morbidity reduction and cost-reduction after SLNB.

## Conclusion

Sentinel lymph node biopsy in patients with bulky squamous cell NVC using ICG-[^99m^Tc]Tc-nanocolloid is feasible, safe and well tolerated with use of local anesthesia. Sentinel lymph nodes were visualized and harvested in all ten patients. Occult metastases were found in five out of ten patients. Further multicenter research is necessary to confirm the generalizability of these findings and investigate the efficacy of SLNB in reducing morbidity and improving oncological outcomes in bulky NVC.

## Supporting information

S1 Protocol Sentinel NOSE study protocol. (PDF)

S2 Table Detailed overview of imaged, yielded and positive sentinel lymph node locations. ^1^According to Wang classification. ^2^According to Robbins and Gregoire. ^3^An unexpected metastasis of an occult concurrent thyroid cancer was found.
(DOCX)
