# Supplementary material for: Sentinel NOSE: Prospective feasibility study on sentinel lymph node biopsy in bulky nasal vestibule cancer
Source: PLoS One. 2025 Jun 13;20(6):e0325764. doi: 10.1371/journal.pone.0325764 (PMC12165422; doi:10.1371/journal.pone.0325764)
Supplement: S1 Protocol — (PDF) [file pone.0325764.s001.pdf]

Sentinel NOSE study

Prospective registration study on the sentinel node procedure for bulky squamous cell carcinoma of the nasal vestibule

**Protocol ID**	**Sentinel NOSE**
**Short title**	**Prospective registration of the sentinel node procedure in bulky squamous cell carcinoma of the nasal vestibule**
**ABR-number**	**70706**
**Version**	**1.1**
**Date**	**15-09-2020**
**Project leader**	**Drs. M.D. Czerwinski**<br>Department of Radiation Oncology<br>Radboud university medical centre<br>PO box 9101<br>6500 HB Nijmegen<br>The Netherlands<br>Telephone: (+31) 06 11079629<br>michal.czerwinski@radboudumc.nl
**Principal investigator**	**Prof. Dr. J.H.A.M. Kaanders**<br>Department of Radiation Oncology<br>Radboud university medical centre<br>PO box 9101<br>6500 HB Nijmegen<br>The Netherlands<br>Telephone: (+31) 024 3614515 / (+31) 06 29501943<br>j.kaanders@radboudumc.nl
**Project members**<br>*(in alphabetical order)*	 **Drs. A. Arens, nuclear medicine physician**<br>Department of Radiology and Nuclear Medicine<br>Radboud university medical centre<br>PO box 9101<br>6500 HB Nijmegen<br>The Netherlands<br>Telephone: (+31) 024 36 13157<br>anne.arens@radboudumc.nl<br><br>**Dr. I. Van Engen-Van Grunsven**<br>Department of Pathology<br>Radboud university medical centre<br>PO box 9101<br>6500 HB Nijmegen<br>The Netherlands<br>Telephone: (+31) 024 36 14314<br>IlsevanEngen-vanGrunsven@radboudumc.nl<br><br>**Dr. R.P. Takes, otorhinolaryngologist / head and neck surgeon**<br>Department of Otorhinolaryngology<br>Radboud university medical centre<br>PO box 9101

	6500 HB Nijmegen<br>The Netherlands<br>Telephone: (+31) 024 36 13508<br>robert.takes@radboudumc.nl<br><br>**Dr. C.G. Verhoef, radiation oncologist**<br>Department of Radiation Oncology<br>Radboud university medical centre<br>PO box 9101<br>6500 HB Nijmegen<br>The Netherlands<br>Telephone: (+31) 024 3614515<br>lia.verhoef@radboudumc.nl<br><br>**Drs. W.L.J. Weijs, oral- and maxillofacial surgeon / head and neck surgeon**<br>Department of Oral- and maxillofacial surgery<br>Radboud university medical centre<br>PO box 9101<br>6500 HB Nijmegen<br>The Netherlands<br>Telephone: (+31) 024 36 93361<br>willem.weijs@radboudumc.nl
**Sponsor**	**Radboud university medical centre**<br>PO box 9101<br>6500 HB Nijmegen<br>The Netherlands<br><br>**Radboud Institute for Health Sciences (RIHS)**<br>Radboud university medical centre<br>PO box 9101<br>6500 HB Nijmegen<br>The Netherlands
**Subsidising party**	**RIHS PI budget (NL: tweede geldstroom)**
**Independent expert (s)**	**Dr. L.G.W. Kerkmeijer, radiation oncologist**<br><br>Department of Radiation Oncology<br>Radboud university medical centre<br>PO box 9101<br>6500 HB Nijmegen<br>The Netherlands<br>Telephone: (+31) 024 3614515<br>linda.kerkmeijer@radboudumc.nl

PROTOCOL SIGNATURE SHEET

Name	Signature	Date
**Head of Department:**<br><br>**Prof. dr. M. Verheij**<br>Department of Radiation Oncology<br>Radboud university medical centre<br>PO box 9101<br>6500 HB Nijmegen<br>The Netherlands<br>Telephone: (+31) 024 3614515<br>marcel.verheij@radboudumc.nl		
**Principal Investigator:**<br><br>**Prof. Dr. J.H.A.M. Kaanders**<br>Department of Radiation Oncology<br>Radboud university medical centre<br>PO box 9101<br>6500 HB Nijmegen<br>The Netherlands<br>Telephone: (+31) 024 3614515<br>j.kaanders@radboudumc.nl		

TABLE OF CONTENTS

1. INTRODUCTION AND RATIONALE 8
2. OBJECTIVES 11
3. STUDY DESIGN 11
4. STUDY POPULATION 11
4.1 Population (base) 11
4.2 Inclusion criteria 12
4.3 Exclusion criteria 12
4.4 Sample size calculation 12
4.5 Sentinel Node biopsy (SNB) 12
5. TREATMENT OF SUBJECTS 13
5.1 Investigational product/treatment 13
6. NON-INVESTIGATIONAL PRODUCT 13
6.1 Name and description of non-investigational product(s) 13
6.2 Preparation and labelling of Non Investigational Medicinal Product 13
7. METHODS 14
7.1 Study parameters/endpoints 14
7.1.1 Main study parameter/endpoint 14
7.1.2 Secondary study parameters/endpoints (if applicable) 14
7.1.3 Other study parameters (if applicable) 14
7.2 Study procedures 14
7.3 Withdrawal of individual subjects 17
7.4 Replacement of individual subjects after withdrawal 17
7.5 Follow-up of subjects withdrawn from treatment 17
7.6 Premature termination of the study 17
8. SAFETY REPORTING 18
8.1 Temporary halt for reasons of subject safety 18
8.2 AEs, SAEs and SUSARs 18
8.2.1 Adverse events (AEs) 18
8.2.2 Serious adverse events (SAEs) 18
8.3 Follow-up of adverse events 19
8.4 [Data Safety Monitoring Board (DSMB) / Safety Committee] 19
9. STATISTICAL ANALYSIS 19
9.1 Primary study parameter 19
9.2 Secondary study parameters 19
9.3 Other study parameters 20
10. ETHICAL CONSIDERATIONS 20
10.1 Regulation statement 20
10.2 Recruitment and consent 20
10.3 Benefits and risks assessment, group relatedness 21
10.4 Compensation for injury 22
10.5 Incentives (if applicable) 23

11. ADMINISTRATIVE ASPECTS, MONITORING AND PUBLICATION 23
11.1 Handling and storage of data and documents 23
11.2 Monitoring and Quality Assurance...24
11.3 Amendments...24
11.4 Annual progress report..24
11.5 Temporary halt and (prematurely) end of study report...24
11.6 Public disclosure and publication policy...25
12. Synthesis...25
13. REFERENCES 26

LIST OF ABBREVIATIONS AND RELEVANT DEFINITIONS

**AE**	**Adverse Event**
**CCMO**	**Central Committee on Research Involving Human Subjects; in Dutch: Centrale Commissie Mensgebonden Onderzoek**
**CV**	**Curriculum Vitae**
**DSMB**	**Data Safety Monitoring Board**
**GCP**	**Good Clinical Practice**
**GDPR**	**General Data Protection Regulation; in Dutch: Algemene Verordening Gegevensbescherming (AVG)**
**HNC**	**Head-and-Neck Cancer**
**IB**	**Investigator’s Brochure**
**IC**	**Informed Consent**
**METC**	**Medical research ethics committee (MREC); in Dutch: medisch-ethische toetsingscommissie (METC)**
**NVC**	**Nasal vestibule carcinoma**
**SPC**	**Summary of Product Characteristics; in Dutch: officiële productinformatie**
**Sponsor**	**The sponsor is the party that commissions the organisation or performance of the research, for example a pharmaceutical company, academic hospital, scientific organisation or investigator. A party that provides funding for a study but does not commission it is not regarded as the sponsor, but referred to as a subsidising party.**
**SPECT**	**Single-photon emission computed tomography**
**SUSAR**	**Suspected Unexpected Serious Adverse Reaction**
**WMO**	**Medical Research Involving Human Subjects Act; in Dutch: Wet Medisch-wetenschappelijk Onderzoek met Mensen**

SUMMARY

Rationale:

Management of the neck in Wang cT1-T2N0 nasal vestibule carcinoma (NVC) has been an ongoing point of discussion. As the disease is rare, publications are scarce, and published regional recurrence rates vary widely between 0% up to 23%. In general, literature recommends adequate radiological neck staging followed by a watchful waiting policy, as overall regional recurrence rates are low (5-10%).
However, according to recent findings, a subset of patients with large or voluminous cT1-T2N0 NVC is deemed at high risk of nodal involvement (20-40% regional recurrence) but receive no elective treatment, although it is well known that presence of nodal metastases impacts the prognosis of head and neck cancer (HNC) dramatically. Whereas elective neck dissection may be too aggressive, sentinel node biopsy (SNB) has been proven a reliable and safe alternative to bridge the gap between imaging and neck dissection.
SNB is currently routinely employed in most HNC centres in the Netherlands and is considered state of the art care, but its application in HNC is limited to oral cavity carcinoma and squamous cell carcinoma of the skin. Following the observation of increased risk of (occult) nodal metastases and regional recurrence in bulky tumors, the sentinel node procedure seems ideally suited for cT1-T2N0 NVC patients. Its superficial tumor localization is easily accessible for peritumoral Tc-99m-nanocolloid-ICG tracer injection. The purpose of this prospective registration study is to document the clinical introduction of the sentinel node procedure for bulky nasal vestibule carcinoma in our centre by protocol, and to identify and address possible unexpected difficulties specific for this tumor site. Ultimately, the goal will be routine and wide implementation of SNB in the NVC subgroup known to be at risk of nodal involvement, as a means to improve regional disease staging and control.

Objective: To prospectively document the introduction of the sentinel node procedure for bulky cT1-T2N0 nasal vestibule carcinoma in patients at risk of nodal involvement.
Study design: Single centre prospective registration study.
Study population: Patients with Wang cT1-T2N0 squamous cell carcinoma of the nasal vestibule, with tumor diameter ≥1.5 cm and/or tumor volume ≥1.5 cm^3, with a WHO performance score of 2 or lower and no history of previous surgery or radiotherapy of the neck.
Interventions: 1. Subcutaneous peritumoral radioactive tracer injection followed by lymphoscintigraphy and SPECT-imaging for sentinel lymph node visualization.
2. Surgical sentinel node biopsy and histopathological examination of harvested nodes following the abovementioned imaging.
Main study parameters/endpoints: The primary endpoint of this study will be successful identification of sentinel nodes on lymphoscintigraphy and SPECT imaging. The procedure will be considered feasible when one or more sentinel nodes can be identified and localized in at least 7 out of the 10 patients..

The secondary outcomes will be: yield of at least one lymph node after biopsy, incidence of surgical complications and pain score during and after peritumoral tracer injection and tracer.

Nature and extent of the burden and risks associated with participation, benefit and group relatedness: The sentinel node procedure is considered state of the art care, but implies additional invasive procedures for the patient. Therefore, proper introduction by protocol and proper documentation are necessary. Prior to radiotherapy, 4 subcutaneous peritumoral injections with radioactive Tc-99m-nanocolloid-ICG will be given at the Nuclear Medicine department, followed by SPECT imaging. Pain may be experienced during tracer injection, which will be scored as one of the study outcomes to assess tolerability. After tracer injection, patients will undergo sentinel lymph node biopsy. They will be at a low risk of minor surgical complications such as postoperative hematoma or infection, and very low risk of major complications, such as nerve damage. There will be additional scarring as a consequence of the surgical procedure. This is offset by possible earlier detection of otherwise occult nodal metastases and a corresponding higher chance of curation by adequate neck treatment. The sentinel node procedure will become the recommended neck staging tool, however, patients will have the option to opt-out of the procedure and hence this study.

1. INTRODUCTION AND RATIONALE

The nasal vestibule is defined as the pear-shaped entrance to the nasal cavity. It is lined with hair-bearing squamous epithelium, and its posterior border is formed by the muco-cutaneous junction named limen nasi. Malignant tumors of the nasal vestibule are therefore almost always squamous cell carcinomas. Squamous cell carcinoma of the nasal vestibule (NVC) is a rare form of cancer, with an incidence of approximately 3 cases per million inhabitants per year [1]. Due to its external location and squamous differentiation, it differs both clinically and biologically from other tumors of the nasal cavity, and therefore requires a distinct treatment strategy. Various staging systems can be employed for NVC, however the Wang classification [2] is considered most suiting for this particular tumor.

Early stage (Wang cT1-T2N0) nasal vestibule cancer can be primarily treated in two ways: surgery or radiotherapy. Tumor control rates for both treatments are comparably high, but cosmetic and functional outcomes can differ dramatically. Since oncological resections and subsequent reconstructions of the nose can significantly impact psychological and social functioning [3], anatomy preserving treatments are preferred when possible. Radiation therapy is an effective anatomy preserving treatment for NVC, avoiding (partial) amputations of the nose. The Radboudumc Centre for Head-and-Neck Oncology (RCHNO) is experienced in interstitial brachytherapy for this tumor. Brachytherapy is a type of internal radiotherapy in which a radioactive source is guided through surgically implanted hollow catheters, allowing a very high tumor dose with limited damage to surrounding tissue. A

recent analysis of image guided brachytherapy results in Wang T1-T2N0 NVC displayed excellent local disease control rates at primary tumor site and patient reported satisfaction [4], which is in line with other reports of this treatment in literature [5, 6].

Although the introduction of image guidance in brachytherapy resulted in excellent local disease control, regional disease control (RC) in neck lymph nodes remains suboptimal. When investigating risk factors for regional recurrence in subgroup analysis, tumor diameter and tumor volume turned out to be prognostically relevant. RC was significantly worse for both tumor diameter ≥1.5 cm (100% vs. 83% when >1.5 cm) and for tumor volume ≥2.3 cm^3 (96% vs. 62% when >2.3 cm^3) [4]. Although these findings have to be validated in larger multicenter series, advanced tumor stage is widely known to be associated with higher risk of nodal involvement [7] and elective neck treatment is universally employed in locally and regionally advanced disease (cT3 and cN1-3). However, management of the neck in Wang cT1-T2N0 NVC has been an issue of ongoing debate. Our observation of early regional failure (one to two years after treatment) is supported by other studies, but as there are only small series published about this disease, regional recurrence rates vary widely between 0% up to 23% [5, 8, 9]. In some series, the 15-20% recurrence rate threshold is approached, at which elective treatment by either irradiation or surgery is recommended [10]. In general, literature recommends adequate radiological neck staging followed by a watchful waiting policy [7], as for the whole group of T1-2 tumors average regional recurrence rates are low (5-10%) and a salvage treatment is available.

Nonetheless, a subset of patients with large, locally non-invasive tumors has a high risk of regional recurrence and receives no additional diagnostics or treatment other than a neck ultrasound, while there is no dispute that presence of even a single nodal metastasis decreases survival in HNC by approximately 50% [7]. Ultrasound-guided fine needle aspiration cytology (US-FNAC) is the current gold standard of neck diagnostics, but with a sensitivity of 86.4% and negative predictive value of 60% [11], it is proven to not be accurate enough to detect all occult regional metastases and replace elective neck treatment in mucosal head-and-neck cancers (HNC) [12].

To bridge the gap between imaging and invasive elective neck dissection, the concept of selective biopsy of the first echelon of draining lymph nodes has been introduced. Now commonly known as sentinel node biopsy (SNB), this procedure has been integrated in standard of care treatment of various tumors. One of the currently widely employed applications of SNB is neck staging of T1-T2 oral squamous cell carcinoma. Recent meta-analyses display excellent negative predictive values up to 90-100% in multiple studies [13]. Similar promising results were found in studies on SNB in high-risk cutaneous squamous cell carcinoma, however these are mostly retrospective and further work is recommended [14, 15]. Furthermore, compared to elective neck dissection, SNB gives significantly less postoperative morbidity and better shoulder function [16, 17]. In the recent years, perioperative SN identification has further been improved by introducing the hybrid fluorescent-radioactive tracer indocyanine green (ICG)–Tc-99m-nanocolloid [18, 19].

In conclusion, SNB is a reliable diagnostic tool to detect occult nodal metastases and improve neck staging, as it is considered to be more precise than solely imaging procedures, and less invasive than elective neck dissection.

A routine sentinel node procedure consists of either subcutaneous or submucosal peritumoral injection of Tc-99m-nanocolloid-ICG, followed by lymphoscintigraphy and single-photon emission computed tomography (SPECT) imaging. Thereafter, sentinel node locations are determined by a nuclear medicine physician and marked on the skin. During SNB on the following day, surgeons use a portable gamma probe and immunofluorescence camera to navigate towards and excise the sentinel nodes. This surgical removal is followed by histopathological examination. The histopathological status determines whether adjuvant therapy is necessary; in case of a tumor positive sentinel node, an additional neck node dissection is performed. When the sentinel node is found to be negative, an observational strategy is justified.

SNB is currently routinely employed in most HNC centres in the Netherlands, but its application in head-and-neck tumors is limited to oral cavity carcinoma and malignancies of the skin. Following our current finding of increased risk of (occult) nodal metastases and regional recurrence, the sentinel node procedure seems ideally suited for cT1-T2N0 NVC patients with bulky tumors. Furthermore, the superficial tumor localization allows access for peritumoral nanocolloid tracer injection. We wish to systematically implement and document the introduction of this procedure to assess any location specific problems associated with the procedure. Ultimately, the goal will be routine implementation of SNB in a select NVC subgroup known to be at risk of nodal involvement, as a means to improve regional disease control.

The purpose of this prospective registration study is to document the clinical introduction of the sentinel node procedure for bulky nasal vestibule carcinoma in our centre by protocol, and to identify possible obstacles or unexpected phenomena in a systematic manner. It is hypothesised that SNB in bulky NVC will prove as feasible as in other head-and-neck tumors, and ultimately will increase regional control rates in patients with large, high risk disease We will prospectively assess the sentinel node procedure from start to follow-up. First, a peritumoral tracer injection and sentinel node SPECT imaging will be done to localize the sentinel node. After successful localization of at least one sentinel node, a subsequent sentinel node biopsy will be performed. All patients with cT1-T2N0 NVC and tumor diameter ≥1.5 cm and/or tumor volume ≥1.5 cm^3 will be eligible for voluntary participation. This cut-off has been established using data from our cohort and [4] significantly discerns patients at high risk of nodal disease involvement. If the procedure is deemed suitable in the first 10 patients, a subsequent larger clinical study will be initiated involving other Dutch centers as well to assess the accuracy of the procedure quantitatively.

2. OBJECTIVES

Primary Objective: To prospectively document the introduction of the sentinel node procedure for bulky cT1-T2N0 nasal vestibule carcinoma in patients at risk of nodal involvement.

Research questions:

1. Are there location specific problems associated with the SN procedure, specifically pain or inability to inject the required nanocolloid volume?
2. Can at least one sentinel node be identified reliably on SPECT after peritumoral nanocolloid injection in patients with bulky T1-T2 nasal vestibule carcinoma?
3. What are common sentinel node locations for nasal vestibule carcinoma?
4. Does the sentinel node procedure yield at least one sentinel node for histopathological analysis?

3. STUDY DESIGN

We will conduct a single centre prospective registration study. Up to ten eligible patients will be accrued from the Radboudumc Centre for Head-and-Neck Oncology (RCHNO). The necessary period of implementation and hence inclusion is estimated at 24 months, due to the low incidence of this disease.

The RCHNO is a nationally acclaimed expertise center for treatment and management of head-and-neck cancer. Approximately 500 new HNC patients are referred yearly to the RCHNO, of which 8-12 are NVC patients. All HNC patients are seen by a multidisciplinary team consisting of medical specialists and paramedics from every involved discipline. The main objective is to deliver excellent personalized curative and palliative treatment, with attention for individual patients and the best functional outcomes.

Furthermore, the RCHNO is one of few centers in the Netherlands with long-standing expertise in nose-sparing brachytherapy treatment of NVC. Therefore, it is ideally suited to conduct this study.

4. STUDY POPULATION

4.1 Population (base)

Up to ten patients with newly diagnosed bulky cT1-T2N0 nasal vestibule carcinoma and planned for curative radiotherapy in the RCHNO will be asked for participation. With 8-12 new cases each year between 2014-2018, an estimated study duration of 24 months is adequate. Study inclusion will be evaluated at 12 months. If inclusion is lagging behind on schedule, either the inclusion period will be prolonged, or expansion to a multicentre cooperation will be established.

4.2 Inclusion criteria

In order to be eligible to participate in this study, a subject must meet all of the following criteria:

- Aged 18 years or older.
- WHO performance score of 0, 1 or 2.
- Newly diagnosed T1 or T2 squamous cell carcinoma of the nasal vestibule.
- Tumor diameter ≥1.5 cm and/or tumor volume ≥1.5cm3
- Clinically negative neck (N0).
- Patients planned to undergo curative treatment.
- Patient provided written informed consent.

4.3 Exclusion criteria

A potential subject who meets any of the following criteria will be excluded from participation in this study:

- Prior allergic reaction to either indocyanide green, 99m-Technetium nanocolloid or human colloidal albumin.
- Pregnancy.
- Previous surgery or radiotherapy of the neck.
- Concurrent secondary head-and-neck tumor.
- Unable to provide informed consent.

4.4 Sample size calculation

Due to the descriptive and explorative nature of this study and endpoints unrelated to clinical outcomes such as disease control or survival, a power calculation has not been performed. An estimated ten patients are deemed necessary to conduct clinical implementation, and eight to twelve cT1-2N0 NVC patients are treated yearly at the Radboudumc, hence the proposed duration of 24 months.

The procedure will be considered feasible when one or more sentinel nodes can be identified in 7 or more out of 10 patients.

4.5 Sentinel Node biopsy (SNB)

Patients with tumor diameter ≥1.5cm and or tumor volume ≥1.5cm^3 are deemed at high risk of regional recurrence according to previous research [4, 20]. Thorough information about sentinel node biopsy and possible therapeutic consequences and risks (see chapters 7.2 and 10.3) will be provided.

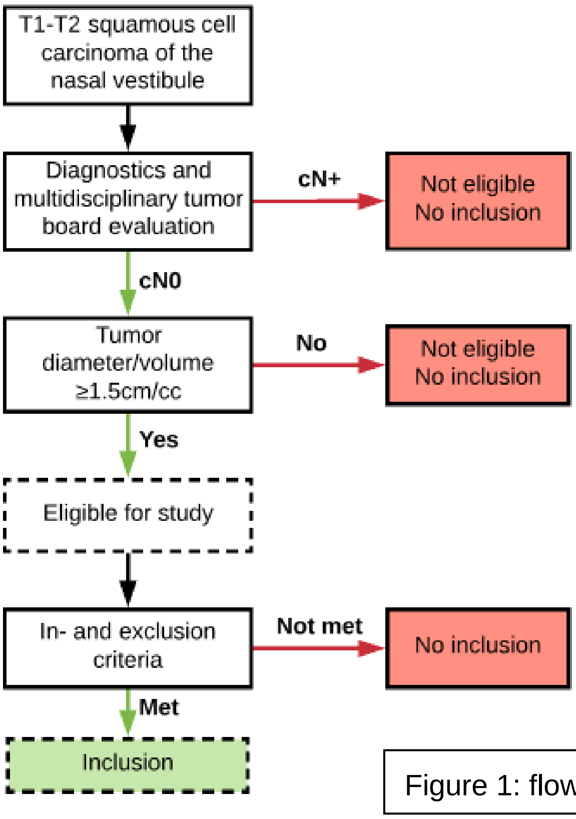

Figure 1: flowchart of inclusion

5. TREATMENT OF SUBJECTS

5.1 Investigational product/treatment

The sentinel node procedure takes place under general anaesthesia. The patient will be intubated orally. When placing the incision, a possible second procedure (neck dissection) is taken into account. The marked sentinel nodes are searched for and removed with the aid of the gamma probe and fluorescence camera. A harmonica drain is left behind. The skin is closed in layers. Gamma probe ten second count and the presence or absence of fluorescence of the removed nodes is noted. The nasal brachytherapy catheters are then placed as usual.

6. NON-INVESTIGATIONAL PRODUCT

6.1 Name and description of non-investigational product(s)

Tc-99m-colloïdaal albumine (Nanocoll)-ICG. Peritumoral depot injections of 20 MBq Tc-99m-nanocolloid-ICG per injection, maximum 4 injections.

6.2 Preparation and labelling of Non Investigational Medicinal Product

Tc-99m-Nanocolloid-ICG is prepared by addition of 50 µl reconstituted ICG solution to a regularly labelled Tc-99m-Nanocoll solution. This exact solution is used in regular clinic practice for the sentinel node procedure in head-and-neck melanoma and oral carcinoma. We refer to the SmPC of Nanocoll and the SmPC of ICG.[21, 22].

7. METHODS

7.1 Study parameters/endpoints

7.1.1 Main study parameter/endpoint

The primary endpoint of this study will be successful and tolerable (for the patient) identification of sentinel nodes on lymphoscintigraphy and SPECT imaging. The procedure will be considered successful and feasible when one or more sentinel nodes can be identified and localized in at least 7 out of the 10 patients. This qualitative endpoint will be assessed by consensus between the nuclear medicine physician (A. Arens), head and neck surgeons (J. Honings, W. Weijs) and radiation oncologists (L. Verhoef, E. Zwijnenburg).

7.1.2 Secondary study parameters/endpoints (if applicable)

The secondary outcomes of this study will be the incidence of adverse events, pain score and analgesic use during and after peritumoral tracer injection, sentinel node yield in biopsy and occurrence of surgical complications after sentinel node biopsy. Adverse events will be scored according to CTCAE 4.0 criteria. Pain will be scored on a 1-10 visual-analogue scale immediately following tracer injection and 5, 10 and 120 minutes after injection. Sentinel node yield will be considered positive if at least one sentinel node can be investigated histopathologically. Finally, surgical complications will be scored according to standard complication registrations forms.

7.1.3 Other study parameters (if applicable)

Patient demographics (age, sex, WHO performance score, smoking status) will be recorded. Furthermore, diagnostic results and baseline tumor characteristics will be registered for all patients. This will include stage, differentiation grade, tumor diameter, tumor volume and tumor epicentre location.

7.2 Study procedures

<u>Nanocolloid injection and SPECT imaging procedures</u>

- Patients will undergo all usual routine diagnostic procedures followed by review in the RCHNO multidisciplinary tumor board. Subsequently, patients will have a follow-up appointment to discuss diagnostic findings and the treatment proposal with their attending physician.
- After this appointment, patients will be informed about the sentinel node procedure and this study by one of the investigators and will be given at least 48 hours for consideration.
- If informed consent is given, patients will be scheduled for tracer injection at the department of nuclear medicine.
    - Sentinel node imaging will be scheduled the day before combined brachytherapy catheter implantation and SNB surgery. It is assured investigational procedures <u>will not</u> delay the start of usual treatment. See also: sentinel node biopsy procedures.

- Subcutaneous nanocolloid injection will be performed by the nuclear medicine physician with HNC sentinel node experience (A. Arens), if necessary consulting the brachytherapy radiation oncologist (L. Verhoef or E. Zwijnenburg) or head and neck surgeon (J. Honings or W. Weijs).
- The injection procedure will be performed according to the existing HNC sentinel node guidelines as follows [23][24]:
    - Patients will be positioned on a bed in a comfortable position. Local anesthesia will be administered and up to 4 peritumoral subcutaneous injections of 20MBq Tc-99m albumin colloid (Nanocoll)-ICG will be given. After injection, the injection location will be dabbed with gauze to remove excess fluid. Gentle pressure with gauze will be applied in case of minor hemorrhage. The anticipated duration of this injection procedure is 1-2 minutes.
- Ten minutes post-injection, static and dynamic scintigraphic imaging will be performed. These images will be taken from anterior and both lateral (left and right) positions. During this, patients will be positioned with the head in light extension using neck support.
- After two to four hours, static imaging from aforementioned positions will be repeated, followed by SPECT of the head-and-neck region.
- All SPECT images will be qualitatively assessed by an experienced HNC nuclear medicine physician (A. Arens) in consultation with the brachytherapy radiation oncologist (L. Verhoef, E. Zwijnenburg) and head-and-neck surgeon (J. Honings, W. Weijs).
    - Sentinel node locations will be also marked on the skin using a gamma probe. Ten second sentinel node emission registered by the gamma probe (so-called count) will be noted. This will be performed by the nuclear medicine physician.
- Either before or after sentinel node localization, patients will be hospitalized to undergo sentinel node biopsy the following day. This implies one additional day of hospitalization.
- The day following SPECT imaging, patients will undergo sentinel node biopsy (for more in depth information, see below), followed by the standard treatment consisting of implantation of brachytherapy catheters under general anaesthesia. Thereafter, they will be hospitalized for seven days the ENT ward. During this time brachytherapy is given two times daily for 14 fractions of 3.5 Gy. After the last fraction is administered, catheters are removed and patients are discharged either on the same or following day.
  The neck drains are removed if the wound fluid production is less than 10cc per 24 hours.

<u>Sentinel node biopsy procedure</u>

- An additional operating time of 2 hours will be scheduled to perform the sentinel node biopsy prior to brachytherapy catheter implantation.
- SNB will be performed prior to brachytherapy catheter implantation according to standard operating procedures in head-and-neck surgery. SNB will always be performed by an experienced head-and-neck surgeon and brachytherapy catheter implantation by a specialized brachytherapy radiation oncologist.
- The SNB will be performed according to the existing HNC sentinel node protocols as described under 5.1.

- After surgery, patients will be admitted at the ENT ward for seven days to undergo brachytherapy. This admission is a standard operating procedure for all NVC patients undergoing brachytherapy as described above.
    - Any harmonica drains left behind after SNB will be removed when drainage is below 10cc/24h; usually after 2 days. Transcutaneous stitches will be removed 7 days postoperatively (on the same day as brachytherapy catheter removal, usually one day before hospital discharge).
- Histopathological examination of the harvested lymph nodes will be performed following biopsy. The results will be reviewed in the RCHNO multidisciplinary tumor board. Eventual additional treatment will depend on this result, as explained below.
    - In case of fully tumor negative sentinel node(s), no additional treatment will be required. Additional staging information in form of N0(sn) will be noted and echo graphic follow-up of the neck shall be employed during follow-up.
    - In case of a positive sentinel node, additional completion neck node dissection will be advised within 3 weeks, according to existing head-and-neck SN guidelines [25].

As sentinel node positivity indicates regional disease extension, without treatment the patient has a high risk of relapse over the course of months, most likely with more extensive disease and possible extracapsular tumor extension. Direct neck dissection in case of positive sentinel node(s) will benefit affected patients. Rates of curation are higher when early elective surgery is employed, compared with therapeutic neck dissection in advanced regional disease [26]. A more comprehensive risk justification is provided in chapter 10.3 and will be discussed with patients prior to inclusion. Since complete neck dissection following tumor positive sentinel node biopsy is standard care and not subject of this research, it will not be explained in detail.

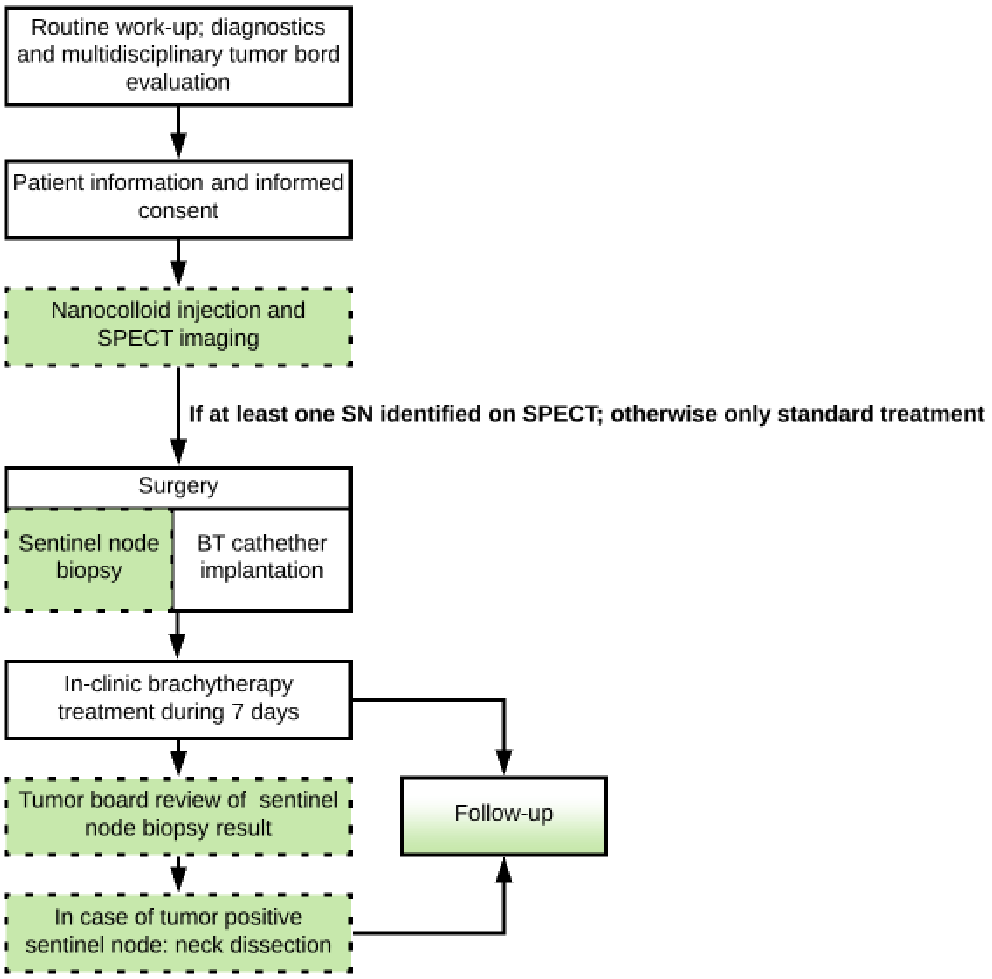

Figure 2: flowchart of procedures; solid line/white background = standard treatment, dashed/green background = investigational interventions.

7.3 Withdrawal of individual subjects

Subjects can leave the study at any time for any reason if they wish to do so without any consequences. The investigator can decide to withdraw a subject from the study for urgent medical reasons.

7.4 Replacement of individual subjects after withdrawal

After withdrawal of a subject, one additional subject will be recruited to achieve the aforementioned ten patient goal.

7.5 Follow-up of subjects withdrawn from treatment

There will be no follow-up of subjects who withdraw from the study, unless the subject's withdrawal was a consequence of urgent medical reasons.

7.6 Premature termination of the study

The study and the clinical introduction of the sentinel node procedure will both be terminated prematurely when unexpected serious adverse events are experienced which may endanger other subjects. If the trial is prematurely terminated the investigator will promptly inform the trial subjects, the medical research ethics committee (METC) and competent authority. A

detailed written explanation of the termination will be provided. Appropriate follow-up and treatment of trial subjects will be ensured.

8. SAFETY REPORTING

8.1 Temporary halt for reasons of subject safety

In accordance to section 10, subsection 4, of the WMO, the sponsor will suspend the study if there is sufficient ground that continuation of the study will jeopardise subject health or safety. The sponsor will notify the accredited METC without undue delay of a temporary halt including the reason for such an action. The study will be suspended pending a further positive decision by the accredited METC. The investigator will take care that all subjects are kept informed.

8.2 AEs, SAEs and SUSARs

8.2.1 Adverse events (AEs)

Adverse events are defined as any undesirable experience occurring to a subject during the study, whether or not considered related to the diagnostic interventions. All adverse events reported spontaneously by the subject or observed by the investigator or his staff will be recorded.

8.2.2 Serious adverse events (SAEs)

A serious adverse event is any untoward medical occurrence or effect that

- results in death;
- is life threatening (at the time of the event);
- requires hospitalisation or prolongation of existing inpatients' hospitalisation;
- results in persistent or significant disability or incapacity;
- any other important medical event that did not result in any of the outcomes listed above due to medical or surgical intervention but could have been based upon appropriate judgement by the investigator.

The elective hospital admission including the aforementioned one planned additional hospitalization day will not be considered serious adverse events.

The investigator will report all SAEs to the sponsor without undue delay after obtaining knowledge of the events, except for the following SAE: prolongation of hospitalization due to brachytherapy related complications (specifically: treatment re-planning due to loss of an interstitial catheter and therefore prolongation of radiotherapeutic treatment). Prolongation of admission caused by surgical/sentinel node biopsy complications will be reported without delay.

The sponsor will report the SAEs through the web portal *ToetsingOnline* to the accredited METC that approved the protocol, within 7 days of first knowledge for SAEs that result in death or are life threatening followed by a period of maximum of 8 days to complete the initial preliminary report. All other SAEs will be reported within a period of maximum 15 days after the sponsor has first knowledge of the serious adverse events.

8.3 Follow-up of adverse events

All AEs will be followed until they have abated, or until a stable situation has been reached. Depending on the event, follow up may require additional tests or medical procedures as indicated, and/or referral to the general physician or a medical specialist. SAEs need to be reported till end of study within the Netherlands, as defined in the protocol.

8.4 [Data Safety Monitoring Board (DSMB) / Safety Committee]

Following EMEA guidelines on Data Monitoring Committees, a DSMB is not deemed beneficial and necessary for this study. Study procedures will be performed by the aforementioned team of experts, in a monitored clinical setting and with a priority for patient safety.

9. STATISTICAL ANALYSIS

As previously explained in Chapter 4.4, a formal power calculation has not been performed due to the explorative nature of this study.

9.1 Primary study parameter

The primary endpoint is successful localization of at least one sentinel node on SPECT imaging for each patient. This endpoint will be assessed by consensus between the nuclear medicine physician (A. Arens), head and neck surgeons (W. Weijs, J. Honings) and radiation oncologist (L. Verhoef, E. Zwijnenburg).
The procedure will be considered feasible when one or more sentinel nodes can be identified and localized in at least 7 out of the 10 patients. This cut-off takes in account a predicted learning curve, which can cause suboptimal results in the first few patients due to lack of experience in sentinel node imaging for this indication.

This qualitative endpoint will be presented in form of categorical data and also visualized as an anatomical map of sentinel node locations corresponding to primary tumor sites.

9.2 Secondary study parameters

Secondary outcomes will be as following: pain score and analgesic use during and after peritumoral tracer injection, incidence of adverse events, sentinel node yield of at least one node after biopsy and incidence of surgical complications.
Adverse events during all study procedures will be graded according to CTCAE 4.0 criteria.

Pain will be scored on a 1-10 visual-analogue scale immediately following tracer injection and 5, 10 and 120 minutes after injection. Any administered analgesics will be documented. Descriptive statistics will be used to present this endpoint.
Yield of at least one lymph node during surgical biopsy is a qualitative endpoint and will be assessed postoperatively by the operating head-and-neck surgeons. In case of successful biopsy, confirmation will be obtained by histopathological examination and subsequent tumor board review. Unsuccessful biopsy will also be reported to the tumor board and discussed after surgery (e.g. no radioactive sentinel node has been found, or biopsy not possible due to risk of damaging vital surrounding structures).

A surgical complication will be defined as any deviation from the normal postoperative course and graded accordingly [27]. Anticipated surgical complications specific to neck SNB are listed in Chapter 10.3.

9.3 Other study parameters

Patient demographics and tumor characteristics mentioned in Chapter 7.1.3 will be used descriptively in tables to indicate the study populations' baseline characteristics.

10. ETHICAL CONSIDERATIONS

10.1 Regulation statement

This study will be conducted according to the principles of the Declaration of Helsinki (9th version, October 2013) and in accordance with the Medical Research Involving Human Subjects Act (WMO).

10.2 Recruitment and consent

To be able to undergo the sentinel node procedure and participate in the study, written informed consent will be mandatory for eligible and willing participants. After diagnostics and multidisciplinary tumor board review, attending physicians will be informed about patient eligibility for the study.
Patients will be recruited by their attending physician during the subsequent follow-up appointment, in which also a treatment plan is proposed. Potential participants will be thoroughly informed about the sentinel node procedure and this study by one of the investigators and will be given at least 48 hours for consideration.
If informed consent is given, patients will be scheduled for tracer injection and SPECT imaging at the department of Nuclear Medicine, and if applicable, for SNB during brachytherapy catheter implantation surgery. It is assured that scheduling and conducting of study procedures will not delay the start of treatment, and that local and national standards of waiting time to surgery are respected.
It will not be possible to undergo the sentinel node procedure during the 24-month implementation period without participation in the prospective registration study.

10.3 Benefits and risks assessment, group relatedness

Patients undergoing the sentinel node procedure and therefore participating in this study will undergo tracer injection and SPECT imaging, followed by surgical sentinel node biopsy. This implies one additional day of hospitalisation, adding up to eight days of hospitalisation including brachytherapy treatment time.

Subjects participating in this study will first undergo Tc-99m-nanocolloid-ICG injection, followed by SPECT imaging. This agent and procedure are already widely implemented in standard care of oral cavity cancer, breast cancer and melanoma, as part of routine diagnostic sentinel node procedure. Hence, the safety of subcutaneous and submucosal nanocolloid tracer injection and the imaging procedure is well documented. Possible side effects for both Nanocoll and ICG are very rare as described in the SPCs, and reported allergic reactions have been mild and manageable so far [28, 29].
Another potential burden may be pain experienced during peritumoral tracer injection in the nasal vestibule. The injection pain is expected to be in line with known cutaneous and mucosal injection pain, and experience shows it to be transient and tolerable. Experienced pain during the procedure is one of the study endpoint and will therefore be monitored and documented. (Non-opioid) analgesics will be administered when necessary to ensure patient comfort.

Lastly, there is a possibility of unexpected findings on the SPECT-scan. This probability is deemed to be very small due to the thoroughness of the diagnostic imaging techniques performed routinely; the area examined by SPECT will not differ from regions of interest scanned routinely. Nonetheless, if the SPECT-scan detects an unexpected finding, this will be communicated to the treating physician and the patient. If patients refuse to be informed about possible unexpected findings they cannot participate and will be excluded. Patients are notified about this by means of the patient information folder and during consultation prior to signing informed consent.

Participation can have beneficial effects for subjects. A tumor negative sentinel node only consolidates N0 neck status and might imply positive prognostic value. However, when a positive sentinel node is found, subsequent completion neck dissection will be advised. Early (elective) neck surgery results in higher survival and disease control rates with lower risk of complications compared to therapeutic neck dissection for regionally recurrent disease [26]. Therefore, it is anticipated that early detection of lymphangeal disease spread (by means of a positive sentinel node) provides a better opportunity for curation by enabling early neck dissection.

Furthermore, this study may have important benefits for nasal vestibule carcinoma patients in the future. If the sentinel node procedure is implemented safely and successfully in our centre, a succeeding larger study will be undertaken, quantitively investigating SNB in patients at risk for occult regional metastases in a multi-centre setting, to establish a sentinel node positivity ratio and to assess whether SNB has a clinically relevant benefit for patients with bulky NV carcinoma. Our ultimate goal is to improve regional disease control and quality of life by early detection and treatment of nodal involvement.

The RCHNO is experienced with sentinel node biopsy of the neck for oral cavity cancer and melanoma. Surgical complications are rare and mostly minor. Most anticipated complications of this procedure are hematoma and wound infection; reported incidence of these complications after SNB is well below 5% [16, 17]. Furthermore, both are well manageable and cause no lasting damage. Depending on sentinel node location, there might be an additional risk of transient neuropraxia of adjacent branches of cranial nerves (e.g. the mandibular branch of the facial nerve or branches of the accessory nerve). However, the reported incidence of this complication is as low as 1% and recovery is almost always complete [16, 17, 30].

One inevitable consequence of the procedure is scarring of the cutaneous incision site. In NV carcinoma, most sentinel nodes are expected in neck levels I-III, but they could also be found in the peri-oral or buccal nodes. Care will be taken to minimize facial scarring during biopsy, however in some cases it may be inevitable. This will be made clear and discussed with eligible subjects before consent, with emphasis on possible cosmetic consequences when peri-oral or buccal sentinel nodes are found.

10.4 Compensation for injury

The sponsor/investigator has a liability insurance which is in accordance with article 7 of the WMO. The sponsor (also) has an insurance which is in accordance with the legal requirements in the Netherlands (Article 7 WMO). This insurance provides cover for damage to research subjects through injury or death caused by the study. The following insurance policies apply:

1. € 650.000,- for death or injury for each subject who participates in the Research;
2. € 5.000.000,- for death or injury for all subjects who participate in the Research;
3. € 7.500.000,- for the total damage incurred by the organization for all damage disclosed by scientific research for the Sponsor as 'verrichter' in the meaning of said Act in each year of insurance coverage.

The insurance applies to the damage that becomes apparent during the study or within 4 years after the end of the study.

10.5 Incentives (if applicable)

Participants will not be paid or reimbursed for participation in this study, yet the opportunity of SNB might be seen as an incentive to participate. We will address this concern during explanation before admission to the study, by emphasizing the scientific nature and yet to be proven successfulness and effectiveness of SNB procedure for nasal vestibule carcinoma.

11. ADMINISTRATIVE ASPECTS, MONITORING AND PUBLICATION

11.1 Handling and storage of data and documents

Scientific studies performed within the department of Radiology and Nuclear Medicine make use of medical imaging data. These imaging data is collected in the context of regular care (reuse of care data) or in the context of scientific research. These image sets are routinely stored in the Radboudumc's Picture Archiving and Communication System (PACS) in the Digital Imaging and Communications in Medicine (DICOM) file format. A DICOM image set consists of images and the DICOM header which stores technical and personally identifiable information. The latter should be removed from the DICOM header before being used for research purposes. Therefore, image sets will be de-identified using the department of radiology and nuclear medicine's DICOM Anonymization Server (DAS). The de-identification process automatically deletes or alters any personally identifiable information in the DICOM header. The resulting de-identified (anonymized) image data sets will be made available to the coordinating investigator and stored as research data suitable for analysis. Only data that is necessary to answer the objective(s) defined in the study protocol of the specific study will be collected.

Members of the radiology and nuclear medicine's trial office perform the de-identification process using only the least amount of personally identifiable information possible (medical record number, year of birth, procedure name and date/accession number/studyID and pseudonym). The year of birth will be used as a check, preventing wrong image data being de-identified as a result of typing errors. This work flow prevents members of the trial office having insight in (additional) personally identifiable information.

Only the study team and the patients attending physicians will have access to the non-anonymized data that are acquired at the department of Radiology and Nuclear Medicine, for the purpose of patient record evaluation. The code list will only contain the patients' initials and surname, date of birth and medical record number and a corresponding pseudonymized code as a key. Pseudonymized information acquired from patients' medical records will be stored in the certified Castor EDC database. Medical confidentiality is applicable to all information acquired at all times. All acquired research data will be stored for a period of 15 years after completing this study. After this period datasets will be destroyed according to applicable law.

11.2 Monitoring and Quality Assurance

In accordance with the risk-based approach for medical research recommended by the Dutch Federation of Academic Medical Centers (NFU), this research is regarded to pose a minor risk to the study population. The RCHNO is experienced with both sentinel node imaging and biopsy, and potential complications and adverse events are well documented. Furthermore, an acceptable WHO performance score of 0 to 2 is required to be eligible for the study. Therefore, we anticipate a low chance of minor damage, and for patients undergoing SNB, a low chance of moderate damage. This imposes a need for minimal monitoring, described as one visit per year with sample monitoring. Detailed monitoring guidelines are displayed in Table 2 of the NFU Handleiding Kwaliteitsborging mensgebonden onderzoek 2.0 [31].

11.3 Amendments

Amendments are changes made to the research after a favourable opinion by the accredited METC has been given. A ‘substantial amendment’ is defined as an amendment to the terms of the METC application, or to the protocol or any other supporting documentation, that is likely to affect to a significant degree:

- the safety or physical or mental integrity of the subjects of the trial;
- the scientific value of the trial;
- the conduct or management of the trial; or
- the quality or safety of any intervention used in the trial.

All substantial amendments will be notified to the METC and to the competent authority. Non-substantial amendments will not be notified to the accredited METC and the competent authority, but will be recorded and filed by the sponsor.

11.4 Annual progress report

The sponsor/investigator will submit a summary of the progress of the trial to the accredited METC once a year, thus in case of this study once. Information will be provided on the date of inclusion of the first subject, numbers of subjects included and numbers of subjects that have completed the trial, serious adverse events/ serious adverse reactions, other problems, and amendments.

11.5 Temporary halt and (prematurely) end of study report

The investigator/sponsor will notify the accredited METC of the end of the study within a period of 8 weeks. The end of the study is defined as the last patient’s last visit.

The sponsor will notify the METC immediately of a temporary halt of the study, including the reason of such an action.

In case the study is ended prematurely, the sponsor will notify the accredited METC within 15 days, including the reasons for the premature termination.

Within one year after the end of the study, the investigator/sponsor will submit a final study report with the results of the study, including any publications/abstracts of the study, to the accredited METC.

11.6 Public disclosure and publication policy

The results of this study will be disclosed unreservedly. Both positive and negative trial results will be disclosed. The results of this research will be submitted for publication to peer-reviewed scientific journals, trial registers, websites or databases.

This trial will be registered in a public trial registry (www.clinicaltrials.gov) before the first patient is recruited [32].

12. Synthesis

Potential issues of concern are not discussed because NANOCOLL Tc-99m-nanocolloid is a registered medical product. Furthermore, Tc-99m-nanocolloid-ICG is already routinely used, both in our clinic and worldwide, for sentinel node sentinel node localization in melanoma, breast cancer and various head-and-neck malignancies such as high risk squamous cell carcinoma of the skin and cancer of the oral cavity [19, 33, 34].

Previously reported adverse effects of this agent have only been mild transient urticarial skin reactions, which in both described cases were easily managed by use of topical corticosteroids [28, 29]. Current consensus concludes that adverse effect are very rare, but nonetheless precautions have to be taken to anticipate upon a possible anaphylactic reaction[22] [21]. For this study, close monitoring of patients during and after nanocolloid administration will be assured. Furthermore, only patients with an acceptable WHO performance score of 0-2 will be included. In case of any signs of adverse reactions, tracer administration will be stopped and appropriate treatment steps will be set in motion in accordance with institution-wide allergic reaction protocols. Concluding, the estimated risk of subcutaneous nanocolloid-ICG injection is low, but adequate preventive measures in form of subject supervision and exclusion of patients in poor condition will be employed nevertheless.

Patients in this feasibility study might benefit from the results of the investigational procedure, whereas a tumor-positive sentinel node will prompt an early curative neck dissection, which may improve disease-free survival.

13. REFERENCES

1. Agger, A., et al., *Squamous cell carcinoma of the nasal vestibule 1993-2002: a nationwide retrospective study from DAHANCA.* Head Neck, 2009. **31**(12): p. 1593-9.
2. Wang, C.C., *Treatment of carcinoma of the nasal vestibule by irradiation.* Cancer, 1976. **38**(1): p. 100-6.
3. Moolenburgh, S.E., et al., *The impact of nasal reconstruction following tumour resection on psychosocial functioning, a clinical-empirical exploration.* Psychooncology, 2009. **18**(7): p. 747-52.
4. Czerwinski, M.D., et al., *Image Guided Brachytherapy for Cancer of the Nasal Vestibule: Local Control and Cosmesis.* Int J Radiat Oncol Biol Phys, 2019. **103**(4): p. 913-921.
5. Levendag, P.C., et al., *Interstitial radiation therapy for early-stage nasal vestibule cancer: a continuing quest for optimal tumor control and cosmesis.* Int J Radiat Oncol Biol Phys, 2006. **66**(1): p. 160-9.
6. Bussu, F., et al., *Comparison of interstitial brachytherapy and surgery as primary treatments for nasal vestibule carcinomas.* Laryngoscope, 2016. **126**(2): p. 367-71.
7. Talmi, Y.P., et al., *Lymph node metastasis in nasal vestibule cancer: a review.* Head Neck, 2011. **33**(12): p. 1783-8.
8. Wray, J., et al., *Radiation therapy for nasal vestibule squamous cell carcinoma: a 40-year experience.* Eur Arch Otorhinolaryngol, 2016. **273**(3): p. 661-9.
9. Vanneste, B.G., et al., *Irradiation of localized squamous cell carcinoma of the nasal vestibule.* Head Neck, 2016. **38 Suppl 1**: p. E1870-5.
10. Weiss, M.H., L.B. Harrison, and R.S. Isaacs, *Use of decision analysis in planning a management strategy for the stage N0 neck.* Arch Otolaryngol Head Neck Surg, 1994. **120**(7): p. 699-702.
11. Stoeckli, S.J., et al., *Initial staging of the neck in head and neck squamous cell carcinoma: a comparison of CT, PET/CT, and ultrasound-guided fine-needle aspiration cytology.* Head Neck, 2012. **34**(4): p. 469-76.
12. de Bree, R., et al., *Advances in diagnostic modalities to detect occult lymph node metastases in head and neck squamous cell carcinoma.* Head Neck, 2015. **37**(12): p. 1829-39.
13. Govers, T.M., et al., *Sentinel node biopsy for squamous cell carcinoma of the oral cavity and oropharynx: a diagnostic meta-analysis.* Oral Oncol, 2013. **49**(8): p. 726-32.
14. Durham, A.B., et al., *Sentinel Lymph Node Biopsy for Cutaneous Squamous Cell Carcinoma on the Head and Neck.* JAMA Otolaryngol Head Neck Surg, 2016. **142**(12): p. 1171-1176.
15. Wu, M.P., R.K.V. Sethi, and K.S. Emerick, *Sentinel lymph node biopsy for high-risk cutaneous squamous cell carcinoma of the head and neck.* Laryngoscope, 2019.
16. Murer, K., et al., *Comparison of morbidity between sentinel node biopsy and elective neck dissection for treatment of the n0 neck in patients with oral squamous cell carcinoma.* Head Neck, 2011. **33**(9): p. 1260-4.
17. Hernando, J., et al., *Comparison of related complications: sentinel node biopsy versus elective neck dissection.* Int J Oral Maxillofac Surg, 2014. **43**(11): p. 1307-12.
18. Brouwer, O.R., et al., *Comparing the hybrid fluorescent-radioactive tracer indocyanine green-99mTc-nanocolloid with 99mTc-nanocolloid for sentinel node identification: a validation study using lymphoscintigraphy and SPECT/CT.* J Nucl Med, 2012. **53**(7): p. 1034-40.
19. KleinJan, G.H., et al., *The best of both worlds: a hybrid approach for optimal pre- and intraoperative identification of sentinel lymph nodes.* Eur J Nucl Med Mol Imaging, 2018. **45**(11): p. 1915-1925.
20. Lipman, D., et al., *Outcome and toxicity profile after brachytherapy for squamous cell carcinoma of the nasal vestibule.* Head Neck, 2015. **37**(9): p. 1297-303.
21. *NANOCOLL Technetium (99mTc) Nanocolloid SPC.* Dutch Medicines Data Bank (Geneesmiddeleninformatiebank CBG), 2018.
22. *VERDYE indocyanidegroen poeder voor oplossing voor injectie SPC.* Dutch Medicines Data Bank (Geneesmiddeleninformatiebank CBG)

2016.

23. Giammarile, F., et al., *The EANM practical guidelines for sentinel lymph node localisation in oral cavity squamous cell carcinoma.* Eur J Nucl Med Mol Imaging, 2019. **46**(3): p. 623-637.
24. Alkureishi, L.W., et al., *Joint practice guidelines for radionuclide lymphoscintigraphy for sentinel node localization in oral/oropharyngeal squamous cell carcinoma.* Ann Surg Oncol, 2009. **16**(11): p. 3190-210.
25. Schilling, C., et al., *Sentinel European Node Trial (SENT): 3-year results of sentinel node biopsy in oral cancer.* Eur J Cancer, 2015. **51**(18): p. 2777-84.
26. D'Cruz, A.K., et al., *Elective versus Therapeutic Neck Dissection in Node-Negative Oral Cancer.* N Engl J Med, 2015. **373**(6): p. 521-9.
27. Dindo, D., N. Demartines, and P.A. Clavien, *Classification of surgical complications: a new proposal with evaluation in a cohort of 6336 patients and results of a survey.* Ann Surg, 2004. **240**(2): p. 205-13.
28. Burton, D.A. and J.N. Cashman, *Allergic reaction to nanocolloid during lymphoscintigraphy for sentinel lymph node biopsy.* Br J Anaesth, 2003. **90**(1): p. 105.
29. Chicken, D.W., et al., *Allergy to technetium-labelled nanocolloidal albumin for sentinel node identification.* Ann R Coll Surg Engl, 2007. **89**(2): p. W12-3.
30. Civantos, F.J., F.L. Moffat, and W.J. Goodwin, *Lymphatic mapping and sentinel lymphadenectomy for 106 head and neck lesions: contrasts between oral cavity and cutaneous malignancy.* Laryngoscope, 2006. **112**(3 Pt 2 Suppl 109): p. 1-15.
31. *Kwaliteitsborging mensgebonden onderzoek 2.0.* Nederlandse Federatie van Universitair Medische Centra (NFU), 2012.
32. De Angelis, C., et al., *Clinical trial registration: A statement from the International Committee of Medical Journal Editors.* New England Journal of Medicine, 2004. **351**(12): p. 1250-1251.
33. Stoffels, I., et al., *Evaluation of a radioactive and fluorescent hybrid tracer for sentinel lymph node biopsy in head and neck malignancies: prospective randomized clinical trial to compare ICG-(99m)Tc-nanocolloid hybrid tracer versus (99m)Tc-nanocolloid.* Eur J Nucl Med Mol Imaging, 2015. **42**(11): p. 1631-1638.
34. Christensen, A., et al., *Feasibility of Real-Time Near-Infrared Fluorescence Tracer Imaging in Sentinel Node Biopsy for Oral Cavity Cancer Patients.* Ann Surg Oncol, 2016. **23**(2): p. 565-72.
