# Supplementary material for: Sentinel NOSE: Prospective feasibility study on sentinel lymph node biopsy in bulky nasal vestibule cancer
Source: PLoS One. 2025 Jun 13;20(6):e0325764. doi: 10.1371/journal.pone.0325764 (PMC12165422; doi:10.1371/journal.pone.0325764)
Supplement: S2 Table — 1According to Wang classification. 2According to Robbins and Gregoire. 3An unexpected metastasis of an occult concurrent thyroid cancer was found. (DOCX) [file pone.0325764.s002.docx]

| No. | Age, sex | Tumor stage^1^ and diameter | Primary laterality  and locations | Imaging sentinel lymph node levels^2^ | Yielded sentinel lymph node levels^2^ | Positive sentinel lymph node levels^2^ |
| --- | --- | --- | --- | --- | --- | --- |
| 1. | 76, M | T2, 2.5cm | Right  Dome+ala+floor | L (cont): Ia (3), Ib (1), II (2), VI (1)  R (ipsi): Ib (1) II (2) | L (cont): -  R (ipsi): Ia (2), Ib (2) | L (cont): 0  **R (ipsi): Ia (1 micro)** |
| 2. | 65, M | T1, 1.5cm | Left  Ala | L (ipsi): Ib (1), II (3)  R (cont): - | L (ipsi): Ia (1), Ib (1), II (0+1 non-SLN)  R (cont): - | L (ipsi): 0  R (cont): - |
| 3. | 66, F | T1, 1.6cm | Bilateral Dome+septum | L: Ib (3), II (2)  R: II (1), VIIa (2) | L: Ib (5), II (1+1 non-SLN)  R: - | L: 0  R: - |
| 4. | 86, M | T2, 2.5cm | Left  Dome+septum+floor | L (ipsi): Ib (2), II (1)  R (cont): Ib (1), II (1) | L (ipsi): II (1), Ib (2)  R (cont): Ib (2 non-SLN) | **L (ipsi): Ib (1 macro)**  R (cont): 0 |
| 5. | 68, F | T2, 3.0cm | Bilateral Septum+floor+philtrum | L: Ib (2), II (1)  R: II (1) | L: Ib (2), II (1)  R: II (1) | L: 0  R: 0 |
| 6. | 75, M | T1, 3.0cm | Left Dome+ala+floor | L (ipsi): Ib (2), III (1)  R (cont): - | L (ipsi): Ib (1+1 non-SLN), III (1)  R (cont): - | **L (ipsi): Ib (1 macro), III (1 thyroïd)^3^**  R (cont): 0 |
| 7. | 52, M | T2, 2.5cm | Bilateral septum Left dome+floor | L: Ib (3)  R: - | L: Ib (1+1 non-SLN)  R: - | **L: Ib (1 macro)**  R: - |
| 8. | 76, M | T1, 1.5cm | Left Septum+floor | L (ipsi): Ib (1), Ia (1)  R (cont): Ib (1) | L (ipsi): Ia (2)  R (cont): Ib (1) | L (ipsi): 0  R (cont): 0 |
| 9. | 60, M | T2, 3.0cm | Bilateral Dome+ala+septum | L: Ib (1)  R: - | L: Ib (1)  R: - | **L: Ib (1 macro)**  R: - |
| 10. | 63, M | T1, 1.5cm | Bilateral septum Left ala | L: Ib (1), VIIa (1)  R: Ib (1) | L: Ia (1)  R: Ib (1+1 non-SLN) | L: 0  R: 0 |

Supplementary Material 2: detailed overview of imaged, yielded and positive sentinel lymph node locations.

^1^According to Wang classification

^2^According to Robbins and Gregoire

^3^An unexpected metastasis of an occult concurrent thyroid cancer was found
